# Supplementary material for: The Slow Progression of Diabetic Retinopathy Is Associated with Transient Protection of Retinal Vessels from Death
Source: Int J Mol Sci. 2023 Jun 29;24(13):10869. doi: 10.3390/ijms241310869 (PMC10341443; doi:10.3390/ijms241310869)
Supplement: Supplementary file 1 [file ijms-24-10869-s001.zip › ijms-2386481-supplementary.pdf]

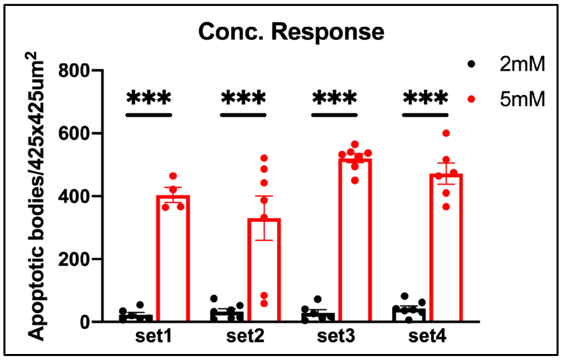

Figure S1 Dose-response to ischemia/ox stress-induced death within retinal vessels. Eyes from non-DM mice were enucleated and treated (insulted) with the indicated dose of TBH for 1 h. Eyes were processed and the extent of death was determined as described in Figure 2. Within a set there were four eyes/group (both eyes from a single mouse and hence 2 mice/group). The student's *t*-test was used to assess if differences between groups were statistically significant; *** $p < 0.005$.

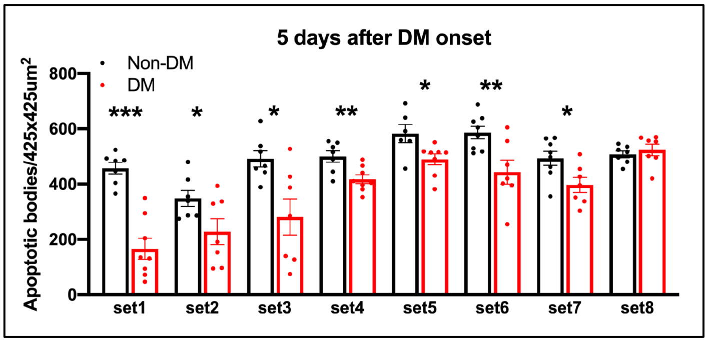

Figure S2 DM induced protection from oxidative stress/ischemia-induced death. The bar graphs show data for each of the 8 sets of eyes that were averaged to obtain the data in Figure 3C. For each of the isolated vascular beds, 6-8 arbitrarily selected regions (425 µm × 425 µm) in the peri-optic nerve zone were selected and photographed. The number of nuclei was counted with Image J; the average for each eye is shown as a dot in the bar graph, which shows the mean ± SEM. The student's *t*-test was used to assess if differences between groups were statistically significant; * $p < 0.05$; ** $p < 0.01$, *** $p < 0.005$.

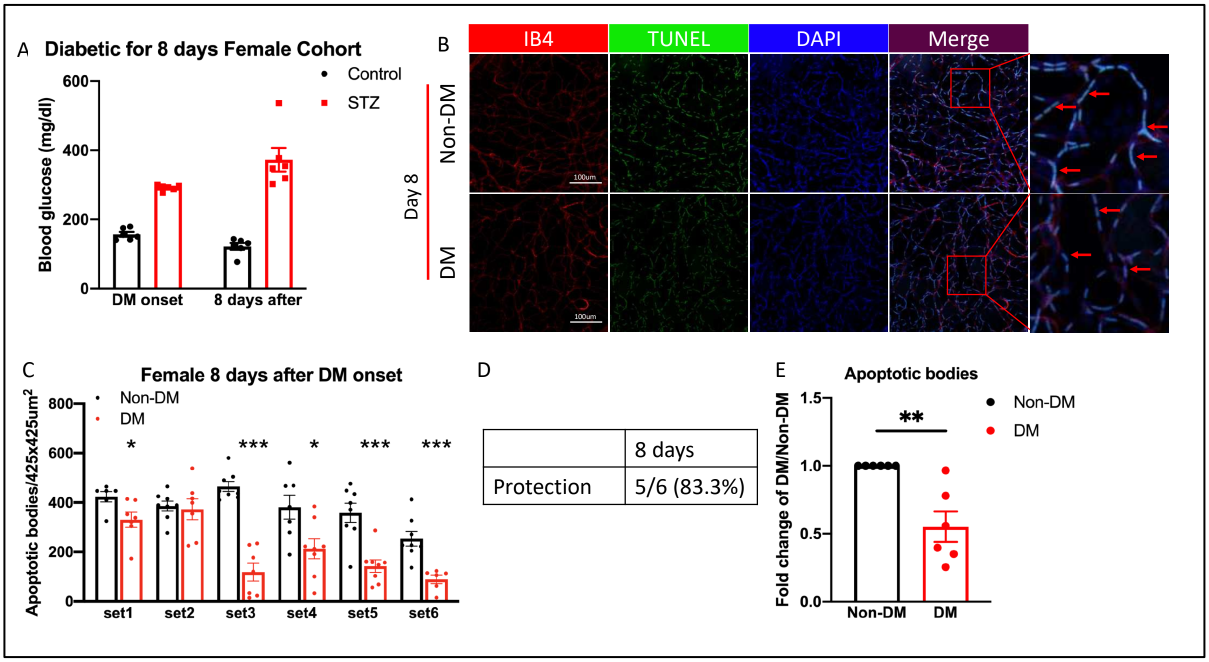

Figure S3 DM induced protection from ox stress-induced death in female mice. **(A)** The average blood glucose at the indicated times. **(B, D and E)** same as Figure 3 except with female mice. **(C)** see legend of Figure S2. * *p*< 0.05; ** *p* < 0.01; *** *p* < 0.005. The red arrows point to representative TUNEL/DAPI double-positive species.

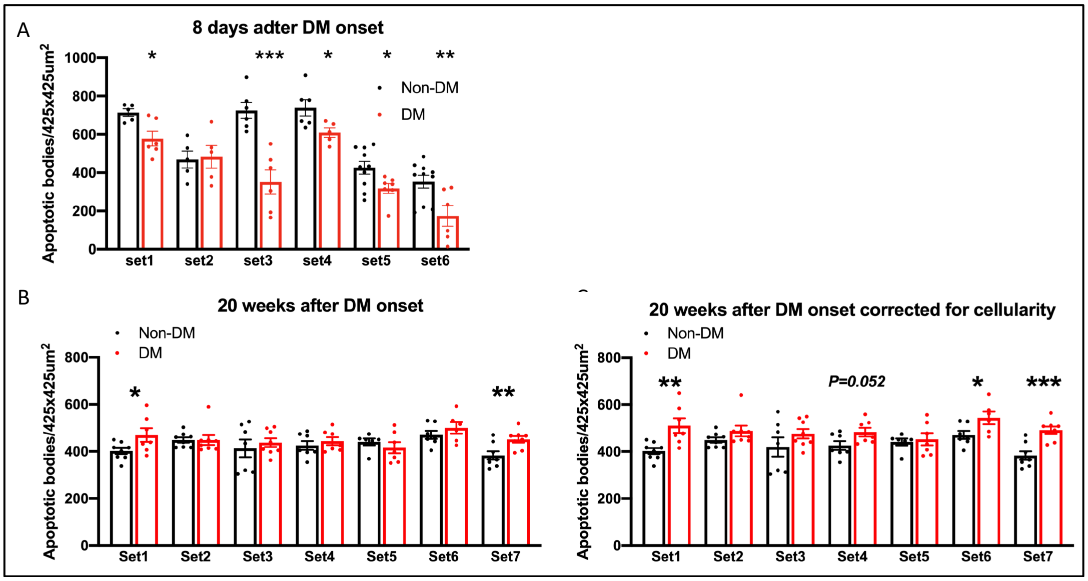

Figure S4 Prolonging the duration of DM was associated with loss of protection and appearance of vulnerability. The bar graphs in panels **(A) and (C)** show data for each of sets of eyes that were averaged to obtain the data in Figure 7B. Panel **(B**) shows the data in panel **(C)** without correction for cellularity. * *p*< 0.05; ** *p* < 0.01; *** *p* < 0.005.

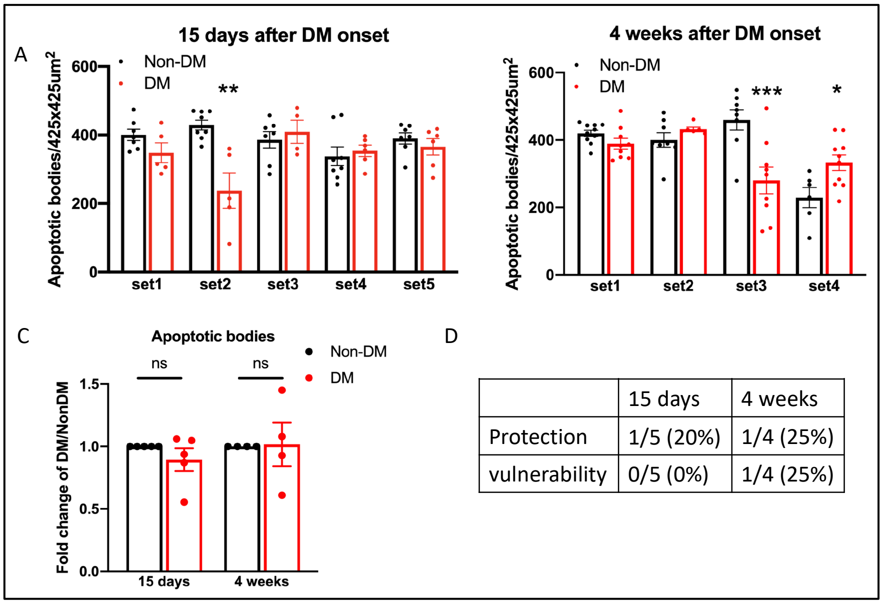

	15 days	4 weeks
Protection	1/5 (20%)	1/4 (25%)
vulnerability	0/5 (0%)	1/4 (25%)

Figure S5 Prolonging the duration of DM was associated with loss of protection and appearance of vulnerability. Panels **(A,B)**: eyes from mice the were DM or non-DM for the indicated duration were insulted with TBH and processed as described in Figure 2; 15 days (n = 5) and 4 weeks (n = 4). For each vascular bed, 6–8 random regions (425 µm × 425 µm) in the peri-optic nerve zone were selected and photographed. The number of oxidative stress/ischemia-induced apoptotic bodies in each region was counted with Image J and represented by a dot in the bar graph. **(C)** The ratio of the average of all of the DM/non-DM sets. **(D)** Table showing protection and vulnerability after the indicated duration of DM. The student's *t*-test was used to assess if differences between groups were statistically significant; * p <0.05; ** p <0.01; *** p < 0.005.

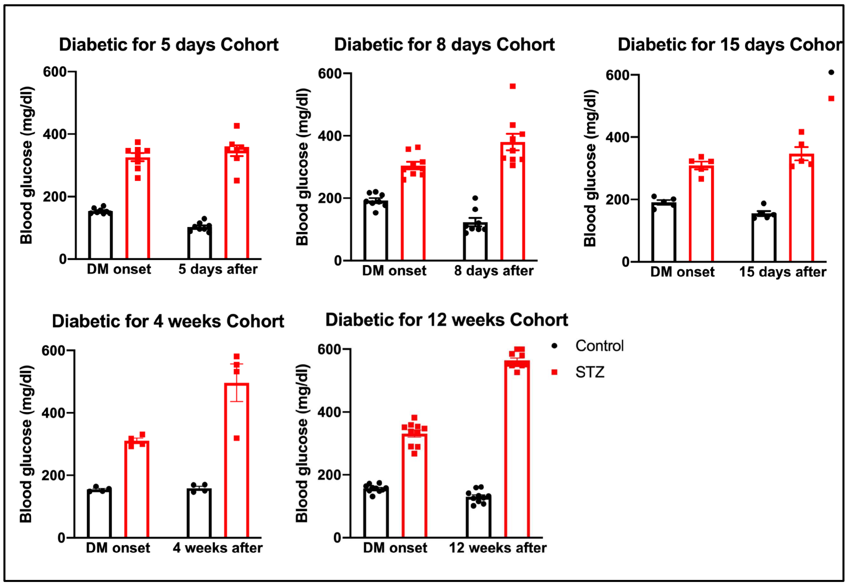

Figure S6 The level of blood glucose in T1D mice. The bar graph shows the average blood glucose at the indicated times; each dot is the value of an individual mouse.

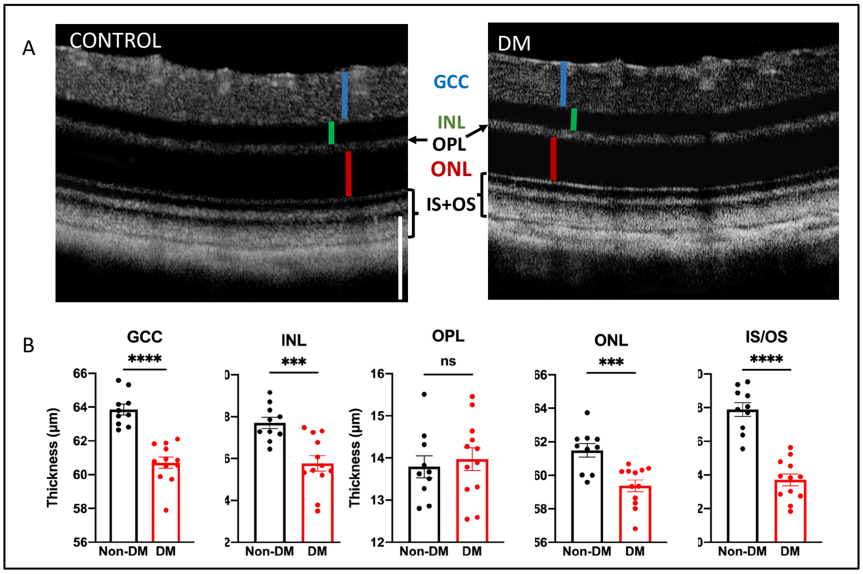

Figure S7 Manifestation of DR was associated with loss of protection and increased vulnerability. **(A)** Representative OCT images of 16 week DM and non-DM mice. **(B)** Quantification of the thickness of the indicated layers of the retina; DM (n = 12) and non-DM (n = 10) mice. *** $p < 0.005$; **** $p < 0.001$.
